# Smart Aging System: Uncovering the Hidden Wellness Parameter for Well-Being Monitoring and Anomaly Detection

**DOI:** 10.3390/s19040766

**Published:** 2019-02-13

**Authors:** Hemant Ghayvat, Muhammad Awais, Sharnil Pandya, Hao Ren, Saeed Akbarzadeh, Subhas Chandra Mukhopadhyay, Chen Chen, Prosanta Gope, Arpita Chouhan, Wei Chen

**Affiliations:** 1 CIME, Department of Electronic Engineering, School of Information Science and Technology, Fudan University, Shanghai 200433, China; 17110720061@fudan.edu.cn (M.A.); rhr1024@sina.cn (H.R.); sd.akbarzadeh@gmail.com (S.A.); ccxx2417@gmail.com (C.C.); arpita2188@yahoo.com (A.C.); w_chen@fudan.edu.cn (W.C.); 2 Computer Science & Engineering Department, Navrachana University, Vadodara, Gujarat 391410, India; sharnil.pandya84@gmail.com; 3 Mechanical/Electronics Engineering, Macquarie University, Sydney NSW 2109, Australia; subhas.mukhopadhyay@mq.edu.au; 4 School of Engineering and Computer Science, University of Hull, Hull HU1 1DB, UK; prosanta.nitdgp@gmail.com

**Keywords:** wellness, elderly, smart home, ambient assisted living, activity of daily living, wellness indices, anomaly detection

## Abstract

Background: Ambiguities and anomalies in the Activity of Daily Living (ADL) patterns indicate deviations from Wellness. The monitoring of lifestyles could facilitate remote physicians or caregivers to give insight into symptoms of the disease and provide health improvement advice to residents; Objective: This research work aims to apply lifestyle monitoring in an ambient assisted living (AAL) system by diagnosing conduct and distinguishing variation from the norm with the slightest conceivable fake alert. In pursuing this aim, the main objective is to fill the knowledge gap of two contextual observations (i.e., day and time) in the frequent behavior modeling for an individual in AAL. Each sensing category has its advantages and restrictions. Only a single type of sensing unit may not manage composite states in practice and lose the activity of daily living. To boost the efficiency of the system, we offer an exceptional sensor data fusion technique through different sensing modalities; Methods: As behaviors may also change according to other contextual observations, including seasonal, weather (or temperature), and social interaction, we propose the design of a novel activity learning model by adding behavioral observations, which we name as the Wellness indices analysis model; Results: The ground-truth data are collected from four elderly houses, including daily activities, with a sample size of three hundred days plus sensor activation. The investigation results validate the success of our method. The new feature set from sensor data fusion enhances the system accuracy to (98.17% ± 0.95) from (80.81% ± 0.68). The performance evaluation parameters of the proposed model for ADL recognition are recorded for the 14 selected activities. These parameters are Sensitivity (0.9852), Specificity (0.9988), Accuracy (0.9974), F1 score (0.9851), False Negative Rate (0.0130).

## 1. Introduction

It is the era of the internet of things (IoT) and ambient intelligence. These technologies have enabled the swift rise of AAL based on smart homes. One of the motivations of smart aging research is the rise of the elderly population. In numerous nations around the world, populations are maturing at an increasing rate, as fertility decays and lifespan rises. As the global population ages, the proportion of people in this cluster is growing. As indicated by a report produced by the United Nations, the extent of people aged 60 years and more, contrasted with the rest of the populace, is projected to double between 2007 and 2050, and achieve 2 billion by 2050 [1].

Deployment of an elderly person into a community care home, particularly when it happens against a person’s desires, has been related with antagonistic impacts, for example, despondency, social detachment, and more prominent reliance on self-care activities. Hence, more elderly people want to remain in their homes instead of entering a medical service or care home when they require specific care services. For those individuals who need exceptional medical attention and monitoring, they may need to be taken out of their comfort zone (i.e., home premises) to meet their requirements. Hospitals, as well as rehabilitation centers, are expensive and unsatisfying. Burden and expenses of care ascend in the medical services framework, as well as for casual parental figures (i.e., relatives, companions, neighbors). The huge expenses related to the arrangement of care for an elderly person, along with deficiencies in the wellbeing workforce, have forced both the caregiving industry (e.g., Elite Care, Intel, Tunstall) and the scholarly world to attempt to explore the adequacy and achievability of wellbeing monitoring and help given in the home [2,3].

In the last decade, various smart home systems have been developed by various researchers. GatorTech was one of the early projects of smart home research at the University of Florida. This was based on ambient sensing units and camera-based monitoring. The CASAS, of Washington State University, started with motion sensing units only in 2007. Recently they have introduced diverse kinds of sensors and actuators. One of the French projects, SWEET-HOME, used audio-input-based monitoring and recognition. This research project revolved more around automation giving control of the home environment to the user through audio-based interaction. In a similar fashion in USEFIL decision support system, they used microphones and cameras for ADL monitoring [2,4,5].

There is a number of ADL-based AAL systems that have been developed to deal with the challenge of elderly wellbeing monitoring. Pulkkinen et al. [6] developed a framework for ADL monitoring for elderly subjects affected by diabetes and from ADL analysis, recognizing the Wellness level. Lara et al. [7] offered a Centinela system, which monitors activity related to gait movements with the application of mobile and other wearable devices. Suryadevara et al. [8] investigated and designed an exceptional system based on heterogeneous wireless sensors networks to detect the behavioral pattern and forecast the anomaly in behavior. Han et al. [9] developed a multi-layered healthcare platform to monitor the activities associated with depression and diabetes, named the long term monitoring research system. There are various context-based models that target the display of context awareness scenarios of AAL. Kim et al. [10] designed an ontology-based context algorithm to offer a healthcare informatics solution. The wellbeing information related to location data, medical information, and activity data in their work was extracted from the wireless sensing system and RFID technology. Lee et al. [11] presented a multiple context-based model that considers subject domain, ambient domain, social context, and function management. This model facilitate personalized healthcare service in the home environment. These systems above lack the feature of knowledge sharing and interoperability between different AAL environment applications.

The appropriateness of activity monitoring for wellbeing status in the smart aging environment has additionally been investigated from the point of view of function with the group of various data sources. McAvoy et al. [12] developed a higher level context framework for indoor monitoring, which covered more or less all significant context-based aspects, such as subject, household object, localization, sub-activity, and time-based features, in a rational and steady style. Zhang et al. [13] offered a method, which considered distributed context conceptions and innovative time-based cognitive aptitudes equally to generate ADL patterns in the AAL. Okeyo et al. [14] proposed an ontological method, which recognized relations between activities and their associated units, and time-based ADL modeling, which introduced relationships among the principal activities of a compound sub-activity.

Steady and precise sensor information is critical for ADLs recognition and classification objectives. Sensor viability, to a large extent, relies upon the action type being perceived. In existing research on activity classification, different kinds of sensors were examined in trials prompting diverse models and formation of the general framework [4,7]. Two principle classifications of sensors can be recognized: Obtrusive (wearable) sensors, and Unobtrusive (non-wearable) sensors. Obtrusive sensors are typically connected to an individual over the body area network (e.g., wristband sensors or cardio sensors) or to their garments (e.g., an accelerometer or a stage counter) to gauge area, beat rate, body temperature, circulatory strain, and other essential measurements or movement attributes. Unobtrusive sensors are typically conveyed in stationary areas of a house or a room and can identify an individual and one´s development and exercises. Unobtrusive sensors can indicate the operational status of household objects as well as subjects, measure water stream, room temperature, or entryway openings and closings. While obtrusive sensors measure precise localization and can recognize body developments and crucial wellbeing characteristics [5,11], unobtrusive sensors are viewed as less intrusive and don’t require any collaboration from the client’s side. Obtrusive sensors may have harsher power utilization requirements. In some of cases these days, the obtrusive sensors may make use of gadgets the client knows about and work with them, for example, a wristwatch or a mobile phone. It is hard to convince someone to wear sensors over the body area network who is healthy. Therefore, the current research work reported in this article is based on hydrogenous unobtrusive wireless sensors networks. These are video-based methodologies in which a camera is introduced in a particular place of a house to identify individual developments, as well as other general exercises. While performing admirably under research facility conditions, this kind of sensor can’t give a similar execution in normal conditions in light of noise interference and background brightness [3,4]. Besides, a camcorder based methodology is viewed as abusive. To address security concerns, a low-resolution thermal imaging sensor was proposed to be utilized rather than a customary camcorder [2,3,4,5]. This sensor can give nearly indistinguishable movement data from a camcorder, while safeguarding the client’s protection.

An assistive living solution is becoming inevitable for lone-living elderly. Many people with incapacities can continue lives in their existing home with the application of assistive informatics and healthcare monitoring. Elderly people’s ability to perform daily activities contribute to the elderly persons Wellness level. Investigation of human activities for extracting health and well-being statistics is one of the present challenges with Smart Aging [15].

The world proportion of the aging population is increasing, and healthcare cost is economically affecting both the general population and governments. When an elderly or disabled individual live with their caretakers, the change in their well-being can be easily detected by caretaker through the change in ADLs, such as behavioral changes related to sleeping duration and use of the toilet [16]. Elderly or disabled persons would even be fine to live with caretakers, but the overloaded of aging population makes it next to impossible to assign caretakers to everyone. To address the elderly’s demand, smart home based AAL systems are the best solution. In 1995, Celler et al. exhibited the first tele-monitoring framework that could decide remotely the functional wellbeing status of older individuals, by consistently and inactively observing connections between old individuals and their living condition over a significant timespan. To achieve this, ADL observation utilized magnetic switches inside the indoor environment that record movement between rooms, infrared sensors on the dividers that distinguish movement in explicit territories of the room, and sound sensors that decide the kind of action. With this improvement, it ended up being conceivable to react prior to behavioral changes, and therefore changes in functional wellbeing status; observed actions would be contrasted with the “typical” behavioral pattern of the individual, and behavioral changes would be noted. The utilization of healthcare monitoring may, thus, provide near real-time analysis that can capture potential emergencies [17].

Smart Home Technology-based AAL offers the monitoring of an inhabitant in a noninvasive manner. The objective of an ambient assistance scenario incorporates smart home technology to enable elderly persons to live independently. Sensor activation-based smart home monitoring keeps caregivers updated about the inhabitant [18,19]. A usual concern for the caregiver is the ability to be immediately alerted and be available in case of any life threating conditions occur to the elderly. For this issue, the smart home system performs monitoring, analysis, and ultimately forecasting to predict serious events for a participant. To forecast the aspects of an individual’s day, designing of intelligent computational approaches is required. Current research work achieves this goal by measuring the ADLs demonstrated by usage of the different household objects, electrical and electronic appliances, and movement inside the home, based on sensor activation.

## 2. Research Challenges and Novelty

Observing the exercises of ADLs and recognition of deviations from past examples is necessary to evaluate the capacity of an older individual to live autonomously in their locale, and in early discovery of unforeseen circumstances. “Smart Ageing” for an older individual is one key component in AAL approach. Daily behavior is a function of the health condition of an inhabitant, and this behavior can be extracted through evaluating the ADLs in terms of different sensory data characteristics, such as timestamp, occurrence, and duration. Once the deviation in the behaviour is identified, the factors causing change can be further analyzed and possibly addressed. Behavioral changes could be classified into three regions: short-term behavioural change, long-term behaviour change, and seasonal behavioural change. Short-term behavioural changes range from a few days to a month, related to activities such as usage of the latrine, which may indicate stomach upset. The long-term behavioural changes are a few months to years, such as an increase in the use of microwave and shower, which may indicate the memory loss that disrupts daily life or more serious forms of Alzheimer’s.

Long-term behavioural changes are the most difficult and complex to detect because it is time-consuming analysis. It is well known that when the human body gets old, its performance degrades physically and mentally; it is even hard for the caretaker to identify the long-term behavioural changes which lead to disease [16]. The caretaker can only request for a routine checkup for further examination. Cooking in a kitchen slowly, and taking a long time for a shower may be dementia or orthopedic weakness (age-related). For long-term behavioural changes, more complex sub-activity needs to be monitored, such as detection of regular fluctuations in the interval of medication or supplements, or missing or repeating medication or supplement dose, may give more accuracy in detecting Alzheimer’s. The short-term behavioural anomaly detection is as easy as comparing with seasonal behavioural change, because the computerized assistive living platform follows time stamp, period, and occurrence [13]. Seasonal behavioural changes occur during the weekend, or during public holidays; these are random days, so it makes it complex to detect the anomaly in behaviour and the causes of an anomaly. Some existing research work based on smart aging either uses a controlled environment (laboratory setup) or uses the manual annotation-feedback. Nonetheless, this performs poorly and fails in the realistic uncontrolled home condition [13,14].

Good health comes from a balanced and disciplined lifestyle. Achieving good health is inevitable for all but ambiguous for many lone-living elderly people. The association between health and daily routine has been investigated and analyzed before, up to a certain extent. With the application of pervasive computing innovations and artificial intelligence, it is possible to enumerate the quality of ADLs and relate daily routine to Wellness. An Assistive Living Home offers elderly Wellness monitoring in the uncontrolled environment. The current proposal monitors every single sensor activation, to extract activities and sub-activities for behavioral pattern detection and recognition to avoid any upcoming unfortunate health risk.

One imperative idea in AAL is the recognition of ADLs. The idea of ADLs is generally utilized in healthcare, abridging exercises and day by day schedules, on which the wellbeing status of an individual is based, and, at last, on which the capacity of an individual to live freely in a network is evaluated. To live independently, an inhabitant is supposed to perform daily activity sets, which include sleeping, bathing, cleaning, eating, entertainment, cooking, dressing, drinking, walking, and taking medication (if one needs). To monitor the set of activities for a lone-living individual, different methodologies have been developed to generate ADL patterns by smart home scientists. ADL modeling is a complex task in the endeavor of smart aging research due to the unique characteristics of activities in the indoor environment. On one side, an assortment of variables ought to be mulled over to portray a sub-activity or activity, for example, localization, the physical article associated with the location, and timestamp [12,16]. These characteristics may change after some time, which makes the ADL recognition increasingly troublesome.

The most common approach is to formulate and detect predefined standard-ADLs that are frequently applied to evaluate the wellbeing of an individual at an abstract level [4,5]. This approach has some assumptions and limitations. On the other hand, every inhabitant is different from one to the other, assuming that they all are going to perform a consistently similar set of predefined ADLs, and in the same manner makes this approach vulnerable (this can be noted as the subject´s interests and preference to conducting an activity). For instance, an occupant who is under monitoring may cook and eat at home daily, defining their routine [19,20]. That same occupant may not cook in the kitchen and start using readymade canned food or eat at friend’s place; this would make monitoring ADLs complex for an AAL. The definitions and declarations of predefined ADL sets are not ideal and fail in the uncontrolled smart home scenario because of interweaved and non-interweaved activities [15]. Recording only preselected activities filters out the other activities, which could provide more details to Wellness evaluation. It is significant to monitor and detect every single activity that an individual performs in their daily routine in the assistive living environment [16,17]. Therefore, an activity might be displayed in different structures that rely upon people’s inclinations and preferences, prompting different ADL variations. The models ought to satisfactorily catch this ADL diversity. As an answer for this, context-aware scenarios are a significant point in our proposed ADL modeling.

Context is characterized as “any data that can be utilized to portray the circumstance of an element. An element is an individual, place, or item that is viewed as important to the connection between a client and an application, including the client and applications themselves” [21]. Zimmermann et al. [22] broaden this definition with five classes of context data: distinctiveness context, timestamp context, localization context, activity context, and associations’ context. This advances the data portrayal of context. In light of this, the context-aware framework utilizes context to give pertinent data and services defined to the client’s assignment. So as to manage the assorted attributes of ADLs, the context-aware framework can be utilized freely with the hardware, operating system, and programming dialect to provide a more prominent scope of gadgets and applications. One complexity of the context-aware scenarios is to deal with and explore the varying ambient environment with another class of uses that know about the context in which they run, and afterward respond to changes in the ambient environment [23].

Since manual evaluation of the ADLs of an individual isn’t plausible in a genuine circumstance, autonomous methodology and monitoring of ADLs utilizing sensors arranged in the indoor home environment is an essential innovation for AAL. ADL monitoring can facilitate the early identification of ailments, for example Alzheimer’s and dementia, and uncover a decline in the capacity of an individual living freely. ADL monitoring produces a few specialized and nontechnical issues that should be tended to. On the specialized technological side, the decision and setup of sensors arranged in home premises, as well as the flag handling and machine-learning calculations to be considered for activity recognition and classification, are imperative. On the other hand, for the nontechnical part, convenience and protection are significant. The most significant and effective framework for ADL monitoring is, therefore, one that requires minimal training datasets or setup exertion and integrates flawlessly into a house. These contemplations represent a few difficulties on the innovation side, including: (a) Sensors must be reasonable, security safeguarding, and simple to introduce and arrange, precluding muddled sensors and receivers—this impacts the feasible ADL classification precision; and (b) information and ground truth procurement for every individual house is exorbitant and arduous. ADL classifiers ought to give sensible execution on an assortment of indoor home environment designs, with extra training datasets as a discretionary contribution to support precision.

Another issue is that the annotation and collection of this much larger data-set is a complex task and introduces latency in smart home monitoring. One of the possible methods is asking inhabitants to give input for their own activity annotation, but this is obstructive, lengthy, and may cause an error during self-tracking [18,19]. The assistive home condition requests event recognition of ADLs from raw sensor information. The sensory activation datasets are mind-boggling and sporadic to encode into predetermined situations. Indeed, even in the wake of encoding this crude information, it is very hard to guarantee the degenerate conduct in light of the fact that these informational indices are on diverse testing rates and sensing modalities [3,5]. These distinctive time and sensor modalities cause inconvenience amid condition definition.

Moreover, within ADL modeling, recognition of ADL change (anomaly detection) is one more decisive and puzzling objective. Anomaly detection is the procedure of identifying behavioural “deviations” of individuals typical routine pattern, and the rare data is exhibited as anomalous behaviour for sorting. This has influenced the concept of the 1-class classification issue, not the old-style 2-class (binary) classification challenge [13,19]. The spotting of fluctuations possibly will comprise the fluctuations in diverse contextual phases, such as location, temporal, time stamp, activity sequence, Wellness level, and more. In principle, behavior (ADLs) uncovering is the concept of displaying an individual’s behavior from sensor activation, whereas anomaly recognition is the concept of differentiating fluctuations in traditional ADLs that also influenced the concept of ADL modeling methods that can successfully identify typical lifestyle traits for variation (anomaly) recognition.

The anomaly detection could be done in two ways: Outlining, and Discerning. Outlining is demonstrating regular behavior and assuming any arriving current input that is dissimilar to the model is an abnormality. Discerning is understanding abnormality data from past data and detecting for a comparable pattern from inward current input data to contemplate an anomaly. Outlining approach is more accurate, as abnormality data is seldom perceived in reality, to offer learning datasets for the classifier [20,22]. Anomaly recognition is in the early stages in smart aging and is more established in other fields, for example, theft uncovering, scam finding, medicinal, manufacturing flaw recognition, image processing, and more. The scope of machine learning methodologies have been utilized for anomaly identification, for example, classification (support vector machine SVM, rule-based, Bayesian,), clustering, statistical (parametric: kernel function, Gaussian; non-parametric: histogram,), nearest neighbor (density based), information theory, and more.

The majority of investigators reported that individual’s events tell more or less “systematic patterns” that can be well-understood from the probabilistic algorithm. Cardinaux et al. [24] suggested a distinct outlining approach with Gaussian Mixture Model (GMM) to form typical data. This method is superior to earlier histogram methods [25,26,27] because GMM might calculate a trait’s reliance. For example, while obtaining both traits of timestamp and period, an action of viewing TV at 3 am (timestamp) for 4 h (period) might be an abnormality. ADLs were mined by the researchers from the sensing data via Rule-Based algorithm, e.g., <Start time, End time, Period>. Some investigators noted that Hidden Markov Model (HMM) is the finest match for the indoor noisy field. As a substitute for applying GMM, Monekosso et al. [28] implemented Hidden Markov Model as a common outlining approach by entering groups of ADLs into the activity framework. They documented that their technique of unsupervised event mining was superior to the traditional supervised technique. Mori et al. [29] selected exploration of the distinct outlining approach by outlining every activities rate of occurrence via probabilistic density technique. However, their methodologies were reported by a number of research fellows for the ineffectiveness of precisely recognizing an anomaly. The reason for the ineffectiveness was two activities could have the same sequence of sub-activities, such as a group of sequential well-ordered sensor activations: “door”, “fridge”, “sink”, “fridge”, “bench”, “fridge”, “microwave”, “door” might denote the action of preparing cereal. Though the similar incident group might signify the action of making coffee or tea, this could be differentiated by the time period of the activities.

Kang et al. [30] explained that Hierarchical HMM is more appropriate than any typical HMM when utilizing the feature of measuring the activity’s period. The researchers considered that the time period of sub-activities in the lower hierarchical level should be lower than the main activity time period, as this main activity is modeled by the two more corresponding sub-activities. However, Chung et al. [31] modified the above by introducing the contextual characteristic of hierarchical configuration through Hierarchical Context–HMM, and applied three HMMs (λSC, λBR, λTR ) in diverse contexts to recognize activities. Duong et al. [32] designed a period model with distinct Coxian distribution and reported that their method is superior in computational cost as compared to any classical technique by unambiguously modeling the period. Their method needed a huge number of parameters for training and testing. Forkan et al. [33] identified two main issues with typical anomaly detection techniques. First, the inability of forecasting upcoming trends that stopped early detection of activities causing disease, and then another, the inclusion of solo context scenarios triggered false alarms for decision making. Subject sickness, e.g., diabetes, is not only because of their mature age, so there is a need to include another context apart from sugar level. Therefore, they integrated HMM with Fuzzy Logic to recognize activities with more than one contextual domain and generate forecasting trends by combining all the information. Wong et al. [34] developed that the finest method to extract profile data is to outline the most frequently visited place of a subject. Novaik et al. [35] applied a Self-Organizing Map to the group and formed activities from sub-activities. Once training is done, any activity that diverges from this group is considered as irregular. However, Wong et al. [34] detected that the system generates an incorrect alarm for abnormality recognition because resident’s ADLs are asymmetrical, such as watching TV until midnight and sleeping long hours in the day time.

This paper proposes a unique anomaly detection method based on a heterogeneous sensing system for elderly people’s ADL and ADL routine monitoring by sensor data fusion. The paper has the following main contributions below. First, proposing an effective sensor data fusion technique based on a wireless heterogeneous sensor network system. The push button indicator is used for recognition of specific daily activities, not as a primary input. The placement information bagged by the room-mounted sensors is utilized for the implication of a user’s room-level ADL. Conferring to the commonly occurring ADLs, the system dexterously split the whole task of identifying all the distinct activities into definite room-based sub-activities. Since each sub-activity categorization model for each sub-event takes recognition of a few activities, the system can improve proficiency and precision. In wireless sensor data fusion, the locality information is necessary to activate the sub-activity models that are pre-trained by the other heterogeneous sensors. Second, in our exploration, we offer a novel attitude to attain significant efficiency from the smart home care framework. To give a reliable arrangement, we proposed and actualized an AAL based on an incorporated system to investigate the person’s conduct on the basis of historical data, real-time freshly received data, and feedback received data. Third, to address the research challenges, we apply behavioral pattern generation through pipeline processing with pre-processing, activity recognition, and smoothing. We focus on isolation of the typical routine information from surprising information, which may cause a risk to Wellness.

We continue the research work by offering the AAL system methodology in Unit III, with the sub-section headings (a) Design and deployment of intelligent wireless sensor and networks (IWSN) (b) Preprocessed Sensor Data Acquisition (PSDA), (c) Activity Annotation, and (d) Wellness Indices Modeling. The investigational results and performance concerns in Unit IV have been represented. The extensions of present research for future work and conclusions with probable solutions towards matter resolution are drawn in Unit V.

## 3. Methodology: AAL Environment Approach and Setup

The framework was planned and actualized in two levels; hardware prototype development, followed by deployment and programming logic. At the prototype development and deployment, the diverse sensing units were developed and positioned to acquire multi-movement and multi-event sensory activation. The present intelligent remote sensing units were designed with Mesh topology, and the crude sensor activation was received by coordinator receiver module and gathered into a server that may be a local home gateway, as well as being cloud-based. In the present research, we have implemented a local home gateway approach for the server. The programming unit was sectioned into various stages, for example, information recording, information mining, and information stockpiling; these modules were responsible for correlating the change in lifestyle with the well-being of an individual in progressive or close time. The present system used a novel Wellness Protocol developed by us. The sensing nodes were intelligent enough to process and transmit the data according to the event and priority of sub-activity [18,19].

**Figure 1:**
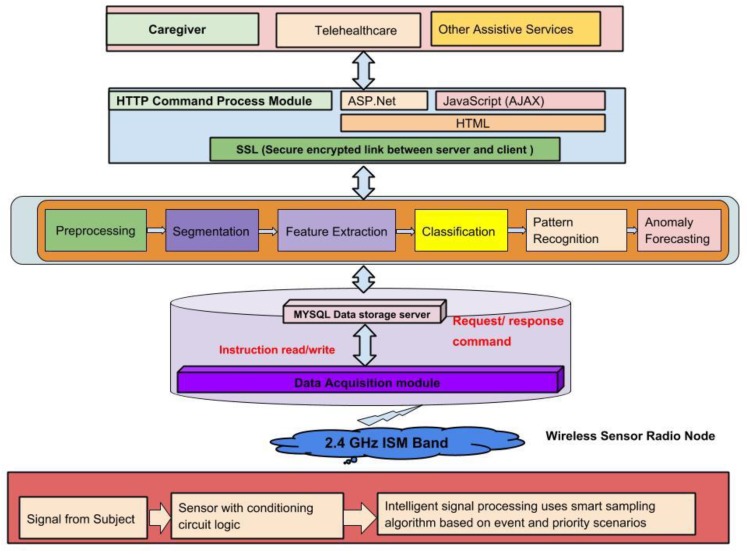
The bottom-up diagram to represent the Smart Home-based AAL system.

Figure 1 depicts the system approach from tiny sensor development to data transmission based on ISM band 2.4 GHz. The diagram shows the flow from data storage into the server to sensor data fusion and decision making information generation. All the node-processed sensory activation data was recorded to represent the events. The present research study offers an innovative sensory data preprocessing and segmentation methodology that combines the sensor physical characteristics based on events, location, and time correlation.

### 3.1. Smart Home Setup Based on IWSN

**Figure 2:**
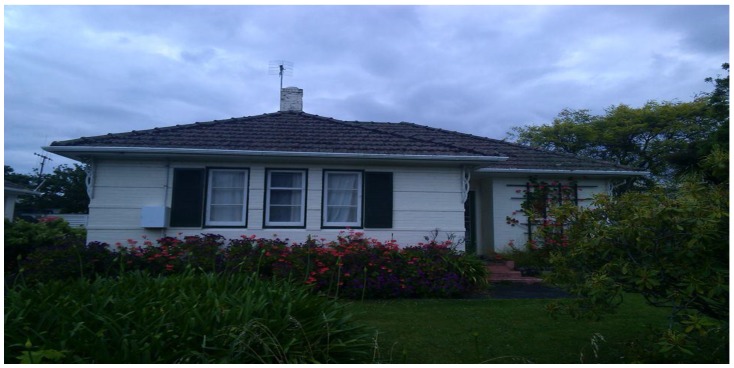
A more than seven-decade-old house where the Smart Aging system was installed without any significant changes in the house.

The present research system named a Wellness system was designed and executed in four different houses. Those houses were the residences of lone-living elderly persons. Figure 2 represents one of the houses where the Wellness-based smart home system was implemented. The Wellness smart home system offers a user-friendly and low maintenance AAL environment, without significant change in interior or exterior of the house. Moreover, it allows an elderly inhabitant to live in an uncontrolled homely condition.

The sensing units are prone to physical damage; apart from that, the system is free from the complexity related to power supply and local home gateway data backup-storage. The algorithm for intelligent pre-processing of sensor activation at the node was developed and implemented on an Intel Galileo Generation-2 baseboard. On the basis of Wellness Protocol approach, sensory activations are transmitted to the local server via radio transceiver [18].

**Figure 3:**
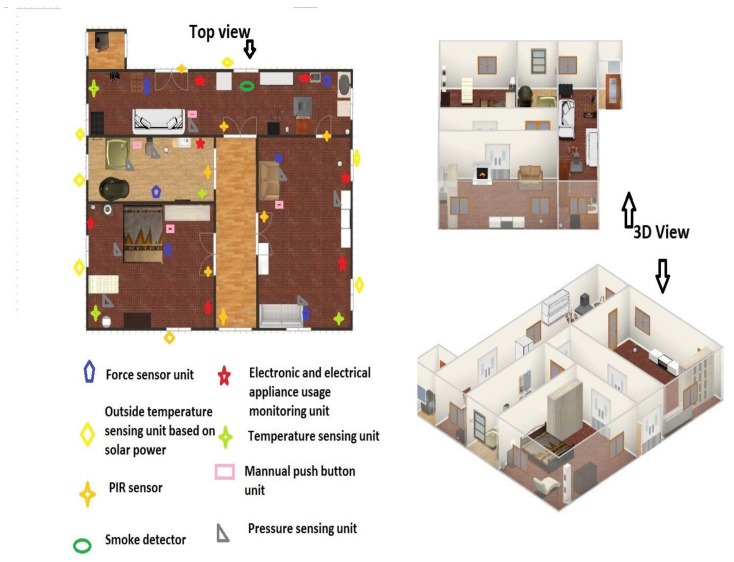
The layout of heterogeneous sensing units’ deployment in the AAL.

The crude sensor activations from sensors (not the sensing unit) are communicated to the baseboard Microcomputer for competent sampling and transmission controller algorithm. The present algorithm analyzes the sensor activations in advance, followed by being applied to Wellness packet encapsulation algorithm. The sampling-transmission control algorithm and packet encapsulation algorithm perform excess data reduction. These algorithms do it through categorization and detection of unavoidable sensor activation from indispensable sensor activation at the sensing unit level before it is communicated to the server. The outline of the AAL environment with sensor positioning is displayed in Figure 3. The sensors are positioned in such a style so that the system gets each indispensable sensor activation commencing in the household, which is valid to the well-being investigation of an elderly inhabitant.

**Figure 4:**
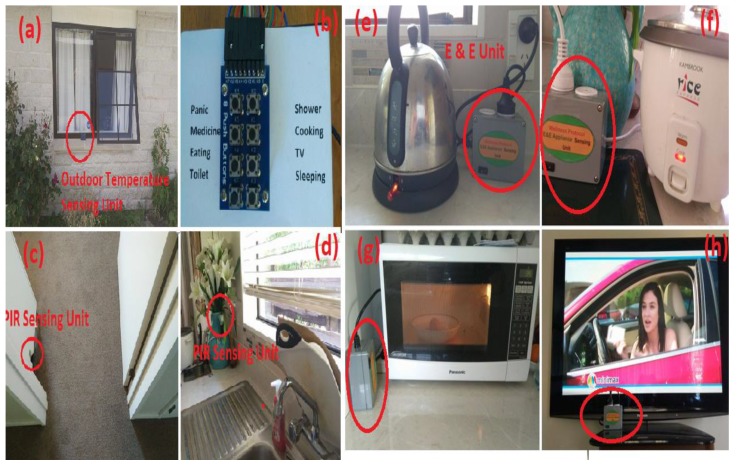
(**a**) Outdoor temperature sensing unit. (**b**) Activity-based manual indication unit. (**c**) Movement sensor deployed at the entry. (**d**) Movement sensor deployed at the corner of the washbasin in kitchen. (**e**) Water cattle plugged into E & E Sensor. (**f**) Rice cooker plugged into E & E Sensor. (**g**) Microwave oven plugged into E & E Sensor. (**h**) Television plugged into E & E Sensor.

**Figure 5:**
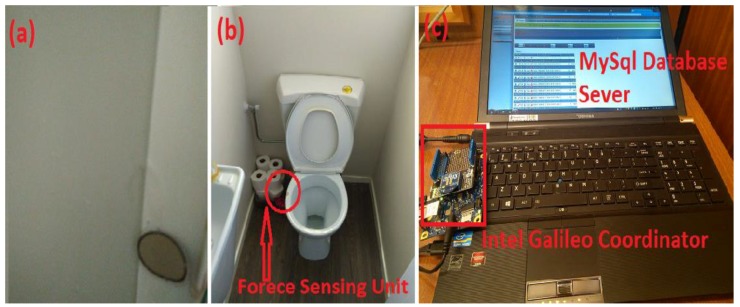
(**a**) Contact sensing to know about the usage of the shower. (**b**) Force sensor deployed over the toilet seat. (**c**) Local Home Gateway Server.

The movement sensors were installed near to doors, windows, corner of rooms, corner of kitchenette, and corner of restroom. Figure 4c represents the Passive Infrared Movement sensing unit deployed at the door and Figure 3. Figure 4d depicts the arrangement of the sensing unit in the kitchen for washing activities. An Electronics and Electrical (E & E) object sensor was plugged into all the electrical and electronic appliances to monitor the time duration of usage. Figure 4e–h represent the E & E unit connected to water cattle, rice cooker, microwave oven, and television, respectively. Figure 5b shows the deployment of the force sensor to measure the frequency of toilet usage and duration. To monitor housing stuff based on the close and open of closet, such as an almirah, office desk, and self-grooming table, wireless contact sensing units were designed. Figure 5b shows the flexiforce A301 used as a contact sensor at the shower door. The frequency of this object usage was monitored by connecting the contact sensing unit at the closet. It is a digital output on/off sensor. Figure 4a shows the outdoor temperature module based on a solar panel deployed at the window.

A push switch key module was a supplementary sensing unit for assessment; the data from it was not considered as primary because of errors during self-input by an elderly person. A manual push button sensor was designed to evaluate and cross-check the performance of activity recognition by the Wellness activity learning model.

### 3.2. Sensor Data Acquisition Module

Present research focuses on indoor ADL recognition for an elderly individual to identify their routine activities and separate the anomaly events. The present system does not directly monitor the toilet usage, hygiene, or bathing, though the system captures how often and how long the inhabitant uses the bathroom from the ambient sensors, contact sensors, and force sensors.

The data collection-related measures are accepted by Massey University Research Ethics Committee and Fudan University Ethics Committee. The sensor data acquisition is done in the elderly subject’s homes based on a heterogeneous wireless sensors and networks. Considering older subjects’ behavior in the function of an uncontrolled realistic environment, the existing elderly houses are converted into smart aging houses. The elderly subjects are encouraged and motivated to self-sufficiently execute each action in their individual way. They are free to go out and take a break from their daily routine. However, for the system, taking a break is also an activity. In the case of data interruption or error/loss, the valid data from the same activity are replaced.

The data collection process considers ninety days as one season of data. The present research work uses 43 weeks data. The present system does not use a defined fixed sampling rate, it uses dynamic sampling. The total average sample size for data is therefore about 16 GB for 2,675,655 sensor activations and 4 subjects. Though the system uses basic data preprocessing, data may comprise overlay, interferences, and noise between activities. The sensor data was raw, but sensory activation was processed before being transmitted from IWSN to Local Server. A Toshiba mobile workstation laptop was used as a Home gateway server. The mobile workstation was a 15.6-inch display, 1TB hard drive, 4 GB Ram, I5 processor. The user interface was developed for the data reception through com-port. Figure 5c shows the Local Server and Intel-board-based coordinator.

### 3.3. Data Pre-Processing by Advanced Belief Model

An era of IoT is where sensing information is linked to the internet. Wireless sensing units collect a huge amount of sensory activation data to apply to machine learning models, and decision or prediction of an event is done on a real-time basis. Nevertheless, the accuracy of ADL monitoring and forecasting is a function of the reliability of sensor data. Unluckily, received sensor data are erroneous and misleading. One of the possible reasons for degradation in sensing data reliability is missing data, repeated data, or erratic data. The missing data, repeated data, and erratic data lead to ambiguities, such as incompleteness, ignorance, and fuzziness. The resource limitations cause missing data. Malfunctioning of the sensing unit brings incompleteness to sensor data.

Thus, the error obstructs the suitable functioning of the smart home monitoring systems and degrades the reliability of data. Consequently, the recognition and detection related to wireless sensing data bring inevitable scrutiny, so it must be processed before being applied to a machine learning system to offer optimum accurate monitoring and forecasting alerts. Hence, it is indispensable to ensure the data reliability and accuracy before feeding it in any data mining and machine learning algorithm.

The majority of sensing units get interference caused by noise or errors; this could be calibrated during system design, though hardware malfunctioning, for example, creating consistent or stationary conduct, should be distinguished. The data received from sensing units are processed as the format of digital and analog series. The sensing unit generates two kinds of qualities, the first is an analog series and the second is a digital series. In the data types due to the atypical (flawed) conduct of sensors, it is possible that they cause underfitting (less information) or overfitting (overabundance information). Therefore, a model has been designed to separate this error, separating data from the inevitable useful data. That model is named as an Advanced Belief Model.

*1. Advanced Belief Model for Analog Data Output*

The analog data, such as temperature value received from a sensor, should be in the modeled characterized run. In the event that it goes past the threshold value or offers a consistent stationary value, at that point there is a high level of belief that shows gadget atypical conduct.

The Wellness threshold for analog-based sensing units is actualized through the linear regression approach. Let us assume, at time instance t, the Advanced Belief figure (BF_t_) is termed through the difference (D_t_) between the derived value (d_t_) and the practical actual value (P_t_) on standard deviation (σ) and ɔ is confidence level (0.95). The derived value function is given by ɖ(t).

(1) $$d_{t}=\frac{1}{N}\sum_{n=0}^{N}ɖ\left(t\right)$$

(2) $${\mathrm{BF}}_{t}=\frac{ɔ}{\left|D_{t}\right|}\;\mathrm{when}\;D_{t}>ɔ$$

Otherwise                  BF_t_ = 0.99  when D_t_ ≤ ɔ (3)

*2. Advanced Belief Model for Digital Data Output*

Digital or discrete valued sensory activations are derived through the Poisson distribution. The Advanced Belief value by Poisson distribution characterizes the probability of occurrence of an occasion in the defined time span. Assume that an independent event to happen “ʖ” times, over a predetermined time interim, at that point the probability of precisely “x” events is equivalent to

(4) $$\mathrm{BF}(x,\;ʖ)\;=\frac{\lambda^{x}e^{-ʖ}}{x!}$$

The Advanced Belief model-based methodology is used for the detection of deviation from the derived value. The modeled value is contrasted with practical value and the distance between them is characterized as residue. The sensory activations are demonstrated by the probability distributions of parameters; data unit, the location of deployment, and time. For precise recognition of deviation, even the smallest residue must be resolved. Accordingly, the observation sampling rate must be sufficiently high for the detection of the lowest residue. By Nyquist Sampling hypothesis, the testing rate should be no less than double the (crest) rate of variation of sensing outputs.

The sensing units are deployed into the realistic home, the home conditions are uncontrolled, so different sources will include noise, for example, RF signals from household object usage. The noise is added to a data signal when the sensor event occurrence time is less than the defined sampling time.

### 3.4. Segmentation

For smoothing of later data mining and machine learning processes, the received analog and digital time series data are segmented into the suitable subwindows. To capture the routine circle of sub-activity, the customized window length according to subject and location of deployment is measured and recorded. The window length was between 14.5 s to 18.2 s for these four elderly subjects (the window specification was 128 and 256 samples) [36]. Additionally, to avoid the useful sensory data loss at the edges of the pair of contiguous sub-windows, the 50% overlap between adjacent sub-windows has been applied.

The total number of window segmentations ɲ for a time series data is given by

(5) $$ɲ=\frac{Ł-Ş}{ł-Ş}$$

where the Ł is the data length, Ş is the overlap size, and ƚ is the segmentation length. The window is split into ɲ sub-windows after the segmentation process.

### 3.5. Activity Modeling

The sensory facts from Wellness packet were extracted by local home gateway loaded with the Wellness sensor data acquisition algorithm. Wellness based activity learning model and activity mining algorithm are applied for pattern detection and anomaly detection.

The key motives of sensor data fusion and Wellness activity modeling are as below:

- Dynamic sensor data fusion: The majority of research work of activity modeling is based on pre-defined sensor datasets. However, the proximate real-time ADL discovery founded on streaming sensory information is left unanswered [4,5,15,16]. Current research work represents an innovative, vibrant real-time sensory event segmentation methodology to offer the solution.
- Dataset collection and variety: Most of the research work around the world does not use heterogeneous unobtrusive sensing in an uncontrolled environment. Moreover, they use one or two home datasets. The present AAL system was deployed in four elderly lone living houses.

In the present research, we had classified the activity detection module containing 3-stages to mine the evidence from sensory activations; the stages were as below:

1. Event Occurrence Stage (1): The present stage comprised all types of sensory stimulation caused by activity accomplished by the elderly inhabitant. The sensory stimulation data was unitless. Thus, it would not generate the behavioral measurements in the current form. The sensor activation records were referred to the higher stage of circumstantial discovery.
2. Circumstantial Factor Mining Stage (2): The circumstantial stage distinguished the elementary activities on the foundation of locality, period, and situation. The elementary activities were directed toward superior stage detection. This stage belongs to the sub-activity data.
3. ADL Recognition Stage (3): The elementary ADLs were categorized and interrelated, dependent on circumstantial figures for pattern discovery.

The present sensor data fusion approach is the heart of the smart aging system, which uses the very first sensory activation information (either 0 or 1) to trigger the subclassification module. The sub-modules are pre-trained by the corresponding heterogeneous sensing data assigned to a specific localization.

For instance, when room “R1” is distinguished as triggered and occupied, then over the next time instance, only the sub module “R1” is initiated and functions as the learning activity. Subsequently, each sub-module is accountable for perceiving fewer ADL compared with the situations of perceiving all the characterized ADL, without applying the entire model of sensor data fusion. By doing this, the activity recognition precision can be enhanced without extra learning. The framework changes to “the entire model” mode to manage the circumstance when there is more than one inhabitant is distinguished, and are caught in the meantime. “The entire model” perceives all the characterized activities together by utilizing the heterogeneous sensing information.

**Figure 6:**
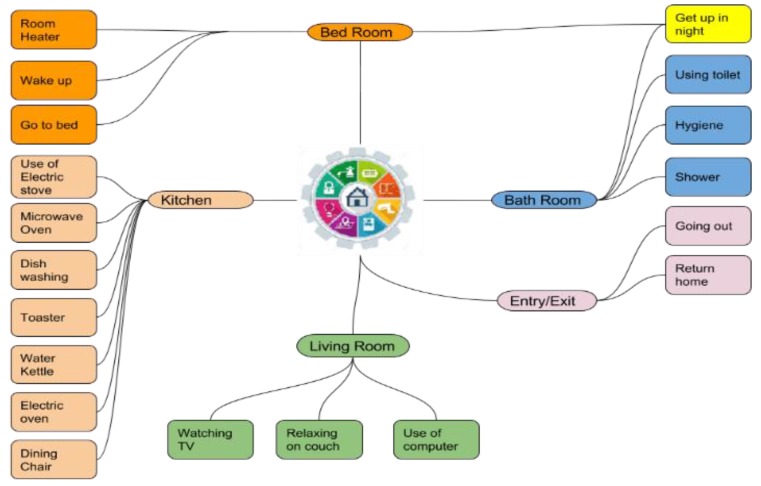
Activities of daily living.

Figure 6 shows a subject’s day by day routine construed from the room-level sensory data of object usage and movements, which can disclose to us when, to what extent, and how regularly the subject remains in particular rooms. Figure 6 similarly gives the points of interest that the individual under monitoring gets up in the room at around the same time in the morning and starts the typical routine, went to bed at the night, and interacts with various household stuff, and so on. Moreover, the room-level everyday routine over quite a while can uncover whether the subject could effectively sort out a day by day life, or whether the subject is driving an irregular routine contrasted with the usual ADL schedule.

**Figure 7:**
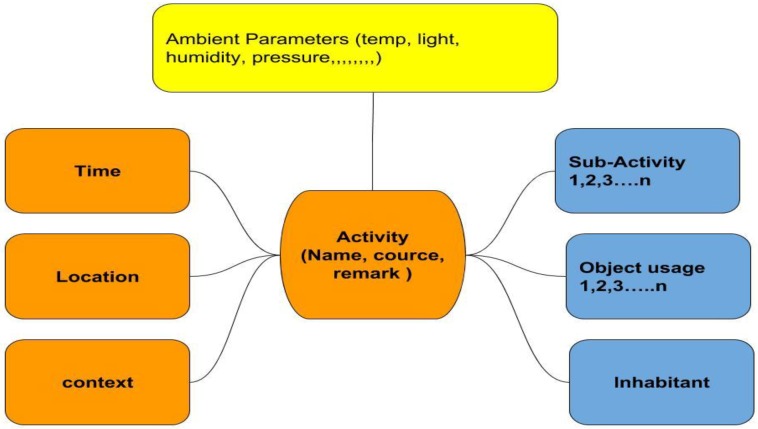
Modeling sub-activity for Activities of Daily Living.

To data mine the information from the heterogeneous sensors, we propose a straightforward, however viable, information combination technique, shown in Figure 6 and Figure 7 and Table 1. The strategy depends on the accompanying presumption: a few activities can be restricted in a particular localization in the view of places of occurrence, e.g., cooking is exceptionally incomprehensible as occurring in a washroom, and teeth brushing may not occur in a room. Here, the subject’s localization information can be utilized to trigger the room-based sub-models. Subsequently, after fusing the first sensor activation of a particular activity to the other most relevant sensory information, the entire classification classifier transforms into parallel-functioning sub-classifiers.

The ADL is extraordinarily unrelated and composite. Thus, merging the diversity of practices, lifestyle, and proficiencies in the course of generating ADLs is problematic. Therefore, the outline of executing the regular routine can be different from one elderly inhabitant to another. In the data-driven attitude of assisted technology-based smart aging feature mining is the unescapable stepladders for behavioral and activity discovery, which mines the evidence from server-stored sensory activations. Such statistics cover the position, period, spell of sensory stimulation, ambient circumstance, the interaction of an individual with a household object, and movement. The activity modeling diagram is shown in Figure 6. An activity is a sequence of sub-activities, for an example, preparing the toast includes taking toast from the refrigerator and placing toast in the toaster, followed by plugging it into the power supply, and washing the dishes in the wash basin. A quadratic sketch of modeling events is shown in Figure 7. The activity labeling and marking for different activities for 24 h is shown in Table 1.

Sensor data fusion has been performed to recognize the sub-activity and ADL by Wellness activity modeling. Table 2 shows the sensor activation collected in the server laptop for four different elderly houses, and the number of ADL recognized successfully from it.

### 3.6. Novel Wellness Indices Modelling and Detection Methodology

Healthcare professionals and caregivers diagnose and support only if the given information is in a user-friendly format, such as a number or well-being index. It is hard for a care provider to understand anything precisely through the position of deployment or node ID of sensory activation. To offer the best possible solution, we designed Wellness grades, Ϣ_1_ and Ϣ_2_. The formulation of Ϣ_1_ was based on non-usage as well as the inactive interval of household stuff or events, whereas the Ϣ_2_ was designed through the usage as well as the active interval of household stuff or events. The Ϣ_1_ and Ϣ_2_ were probabilistic functions. Therefore, when Ϣ_1_ and Ϣ_2_ were equivalent to 1, the elderly inhabitant was well.

The functions Ϣ_1_ and Ϣ_2_ were the BASIC indices, those were later upgraded through the inclusion of multiple observations of seasonal deviations and random days. The upgrading remarkably reduced the false positive alert massages [6]. In the upgraded Wellness function, the seasonal deviation observations had been familiarized by the time series modeling. The below offered method in equations was introduced by dynamic sensor data fusion and time series principle.

(6) Ϣ1=e−tTIN

where Ϣ_1_ well-being function was formulated over the inactive spell duration of household use by the elderly inhabitant; t was the definite spell duration of idleness (inactive) of all or specific household; *T_IN_* was ultimate- idleness interval and outside this may grounds abnormality situation that duration was calculated from past observations. For the regular life of an individual Ϣ_1_ should be 1.

(7) $${Ϣ}_{2}=e^{\frac{\left(T_{N}-T_{A}\right)}{T_{n}}}$$

where Ϣ_2_ Wellness function was formulated over the active spell duration of household use by the elderly inhabitant; *T_A_* was the definite spell duration of activity of all or specific household; *T_N_* was an ultimate-active interval and outside this may be the grounds for an abnormality situation. That duration was calculated from past observations. For the regular life of an individual, Ϣ_2_ should be 1. The abnormality is only calculated when *T_A_* > *T_N_*. The regular duration of household use of an individual specifies how effectively one performs everyday indoor work. Conferring to cyclical variations, the elderly individual’s behavior and the spell duration of object use vary but are not considered an abnormality situation. It should not be designated as a cautionary alert. For instance, the elderly individual’s daily activities would not be similar from rainy to winter season; there will be a substantial transformation in the activities. Therefore, a behavioral adjustment must be recognized and detached from the abnormality symptom. For the precise evaluation of routine change and anomaly discovery, *T_IN_* and *T_N_*, time of household idleness and use, respectively, were framed according to the calendar and cyclical variations. Time series machine learning practices were applied to develop the statistical approach for *T_IN_* and *T_N_*.

Characterization of the pictogram utilized in formulations starting from Equations (8)–(20):

L = numeral of the interval in a single sequence or cyclical span;
α = Statistics smoothing element 0 to 1;
γ = Cyclical variation smoothing element 0 to 1;
x = Note of object at the present spell;
S = Smoothed observation;
T_t_ = Tendency factor;
C = Cyclical Drift;
F = The prediction at “m” episodes in the future;
t = Key to display the spell duration;
m = numeral of stages in advance of prediction;
Ϣ = Trend smoothing factor 0 to 1.

The smoothed observation for ultimate idle household use was assessed by the recent times recorded sensory episodes $\left\{\alpha \;\left(x_{t}\;-C_{t-L}\right)+\left(1-\alpha \right)\left(S_{t-1}-T_{IN\;t-1}\right)\right\},$ and the episodes happened throughout the matching term in preceding year r $\left\{\alpha^{\prime }\left(x_{t}^{\prime }-C_{t-L}^{\prime }\right)+\left(1-\alpha^{\prime }\right)\left(S_{t-1}^{\prime }+T_{IN\;t-1}^{\prime }\right)\right\}$.

(8) $$S_{t}=\frac{1}{2}\left[\left\{\alpha \;\left(x_{t}\;-C_{t-L}\right)+\left(1-\alpha \right)\left(S_{t-1}-T_{IN\;t-1}\right)\right\}+\;\{\alpha^{\prime }\left(x_{t}^{\prime }-C_{t-L}^{\prime }\right)+\left(1-\alpha^{\prime }\right)\left(S_{t-1}^{\prime }+T_{IN\;t-1}^{\prime }\right)\}\right]$$

The ultimate inactive household practice was assessed by the recent times recorded sensory episode factors $\left\{\beta \;\left(S_{t}\;-S_{t-1}\right)+\left(1-\beta \right)\left(T_{IN\;t-1}\right)\right\}$, and the episode arose throughout the matching term in preceding the year $\left\{\beta^{\prime }\left(S_{t}^{\prime }-S_{t-1}^{\prime }\right)+\left(1-\beta^{\prime }\right)\left(T_{IN\;t-1}^{\prime }\right)\right\}$.

(9) $$T_{IN}{\;}_{=}\;\frac{1}{2}\left[\left\{\beta \;\left(S_{t}\;-S_{t-1}\right)+\left(1-\beta \right)\left(T_{IN\;t-1}\right)\right\}+\;\{\beta^{\prime }\left(S_{t}^{\prime }-S_{t-1}^{\prime }\right)+\left(1-\beta^{\prime }\right)\left(T_{IN\;t-1}^{\prime }\right)\}\right]$$

The cyclical trend for ultimate inactive household practice was assessed by the recent times recorded sensory episode factors $\left\{\gamma \;\left(x_{t}-S_{t}\right)+\left(1-\gamma \right)\left(C_{t-L}\right)\right\}$, and the episode arose throughout the matching term in the preceding year $\left\{\gamma^{\prime }\left(x_{t}^{\prime }-S_{t}^{\prime }\right)+\left(1-\gamma^{\prime }\right)\left(C_{t-L}^{\prime }\right)\right\}$.

(10) $$C_{t}=\frac{1}{2}\left[\left\{\gamma \;\left(x_{t}-S_{t}\right)+\left(1-\gamma \right)\left(C_{t-L}\right)\right\}+\;\{\gamma^{\prime }\left(x_{t}^{\prime }-S_{t}^{\prime }\right)+\left(1-\gamma^{\prime }\right)\left(C_{t-L}^{\prime }\right)\}\right]$$

The smoothed observation for ultimate active household use was assessed by the recent times recorded sensory episodes $\left\{\alpha \;\left(x_{t}-C_{Nt-L}\right)+\left(1-\alpha \right)\left(S_{Nt-1}-T_{N\;t-1}\right)\right\},$ and the episodes happened throughout the matching term in preceding the year $\left\{\alpha^{\prime }\left(x_{t}^{\prime }-C_{Nt-L}^{\prime }\right)+\left(1-\alpha^{\prime }\right)\left(S_{Nt-1}^{\prime }+T_{N\;t-1}^{\prime }\right)\right\}$.

(11) $$S_{Nt}=\frac{1}{2}\left[\left\{\alpha \;\left(x_{t}-C_{Nt-L}\right)+\left(1-\alpha \right)\left(S_{Nt-1}-T_{N\;t-1}\right)\right\}+\;\{\alpha^{\prime }\left(x_{t}^{\prime }-C_{Nt-L}^{\prime }\right)+\left(1-\alpha^{\prime }\right)\left(S_{Nt-1}^{\prime }+T_{N\;t-1}^{\prime }\right)\}\right]$$

The ultimate active household practice was assessed by the recent times recorded sensory episode factors $\left\{\beta \;\left(S_{Nt}-S_{Nt-1}\right)+\left(1-\beta \right)\left(T_{N\;t-1}\right)\right\}$, and the episodes happened throughout the matching term in preceding the year $\{\beta^{\prime }\left(S_{Nt}^{\prime }-S_{Nt-1}^{\prime }\right)+\left(1-\beta^{\prime }\right)\left(T_{N\;t-1}^{\prime }\right)$.

(12) $$T_{N}=\frac{1}{2}\left[\left\{\beta \;\left(S_{Nt}-S_{Nt-1}\right)+\left(1-\beta \right)\left(T_{N\;t-1}\right)\right\}+\;\{\beta^{\prime }\left(S_{Nt}^{\prime }-S_{Nt-1}^{\prime }\right)+\left(1-\beta^{\prime }\right)\left(T_{N\;t-1}^{\prime }\right)\}\right]$$

The cyclical trend for ultimate active household practice was assessed by the recent times recorded sensory episode factors $\left\{\gamma \;\left(x_{t}\;-S_{Nt}\right)+\left(1-\gamma \right)\left(C_{Nt-L}\right)\right\},$ and the episodes happened throughout the matching term in preceding the year $\left\{\gamma^{\prime }\left(x_{t}^{\prime }-S_{Nt}^{\prime }\right)+\left(1-\gamma^{\prime }\right)\left(C_{Nt-L}^{\prime }\right)\right\}$.

(13) $$C_{Nt}=\frac{1}{2}\left[\left\{\gamma \;\left(x_{t}\;-S_{Nt}\right)+\left(1-\gamma \right)\left(C_{Nt-L}\right)\right\}+\;\{\gamma^{\prime }\left(x_{t}^{\prime }-S_{Nt}^{\prime }\right)+\left(1-\gamma^{\prime }\right)\left(C_{Nt-L}^{\prime }\right)\}\right]$$

F_*t*+m_ = *S_t_* + m*T_IN_* + *C*_*t-L* + 1 + ((m−1) mod L)_ (14)

F_*Nt*+m_ = *S_Nt_* + m*T_N_* + *C*_*Nt-L* + 1 + ((m−1) mod L)_ (15)

(16) $$S_{t}=\frac{1}{L}\;\left(x_{1}+\;x_{2}+\;x_{3}+\;x_{4}\ldots \ldots x_{L}\right)$$

(17) $$S_{Nt}=\frac{1}{L}\;\left(x_{1}+\;x_{2}+\;x_{3}+\;x_{4}\ldots \ldots x_{L}\right)$$

(18) $$C_{1}=x_{1}-\;S_{t},\;C_{2}=x_{2}-\;S_{t}$$

(19) $$CN_{1}=x_{1}-\;S_{Nt},\;CN_{2}=x_{2}-\;S_{Nt}$$

(20) $$T_{t}=[(\frac{1}{L})\{(\;\frac{x_{L+1}-x_{1}}{L})+(\frac{x_{L+2}-x_{2}}{L})+\;(\frac{x_{L+3}-x_{3}}{L})+\;\ldots \ldots +\;(\frac{x_{2L}-x_{L}}{L})\}]$$

The conduct of the elderly inhabitant was characterized as regular or anomaly, depending on the equations derived. For the forecasting, the present activity duration was correlated with the forecasted duration. The confidence level of 95% was presumed for the prediction analysis. The acceptable limit of the period for any routine action was defined by Equation (21) below. If the actual duration was beyond the limit according to Equation (21), then an anomaly flag was triggered.

Period of routine action = Prediction period ± 2*standard deviation
(21)

### 3.7. Classification and Performance Assessment

For classification issues, the methodology measures the implementation of a model as far as its error rate—level of erroneously classified occasions in the dataset. The present system manufactures a model since it needs to be utilized to arrange the new information. Henceforth, we are predominantly intrigued by model accuracy on new (concealed) information. We utilized two informational collections: the training set (seen information) to construct the model (decide its parameters), and the test set (concealed information) to measure its execution and performance (holding the parameters consistent). Usually, the system needs the validation set to tune the model. The validation set should not be utilized for testing (as it’s not inconspicuous or hidden). Every one of the three informational datasets must be illustrative examples of the information that the model will be connected to.

The accessible informational dataset from all subjects are divided into 10 approximately equal size folds, and each fold generally has the same number of samples from every ADL of each subject. Seven folds are utilized as training information, one fold serves for cross-validation, and two-folds are for testing the model. Every one of the 10 folds is utilized precisely once as test information, and the test information is inconspicuous for the classifier. The outcomes revealed in the remainder of the paper are based on 10 test measures.

To validate the proposed algorithm, there are four possible results

For ADL:
  1. True positive (TP): The “A” activity happens and the algorithm detects it correctly the “A” activity.
  2. False positive (FP): The “A” activity does not occur and the algorithm reveals the “A” activity.
  3. True negative (TN): a daily event is performed and the algorithm does not detect it in the “A” activity.
  4. False negative (FN): The “A” activity happens but the algorithm does not detect it.
For anomaly:
  1. True positive (TP): an anomaly happens and the algorithm detects it.
  2. False positive (FP): an anomaly does not occur and the algorithm reveals an anomaly.
  3. True negative (TN): a daily event is performed, no anomaly occurs, and the algorithm does not detect it.
  4. False negative (FN): an anomaly occurs but the algorithm does not detect it.

The performance of the system can be evaluated considering the following metrics based on confusion matrix:

Sensitivity (Recall) = TP/(TP+FN)
(22)

Specificity = TN/(TN+FP)
(23)

Precision = TP/(TP + FP)
(24)

Negative Predictive Value (NPV) = TN/(TN + FN)
(25)

False Positive Rate (FPR) = FP/(FP + TN)
(26)

False Discovery Rate (FDR) = FP/(FP + TP)
(27)

False Negative Rate (FNR) = FN/(FN + TP)
(28)

Accuracy (ACC) = (TP + TN)/(P + N)
(29)

F1 Score (F1) = 2TP/(2TP + FP + FN)
(30)

Correlation Coefficient= TP*TN − FP*FN/sqrt((TP+FP)*(TP+FN)*(TN+FP)*(TN+FN))
(31)

## 4. Experimental Analysis, Observations, and Comparative Arguments

Based on the experiments performed in four different elderly lone-living houses over the three hundred and one days, the ultimate inactive period and ultimate usage period of household objects could be calculated, through the provisional implementation duration of the Wellness platform. The trial duration is the function of an individual’s well-being to perform ADL. Once the Wellness Time Series Model acquires the behavior of the day-to-day activities, at that point the provisional execution stage would be upgraded to the testing stage to evaluate the Wellness indices.

**Figure 8:**
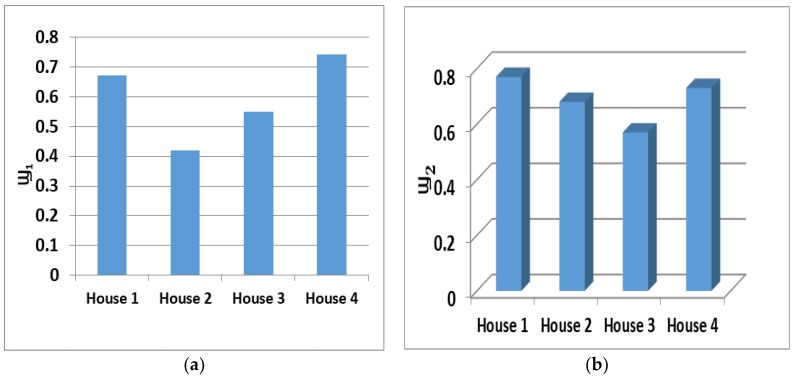
(**a**) The (Basic) Ϣ_1_ for four different houses up to one week. (**b**) The (Basic) Ϣ_2_ for sleeping activity for four different houses up to one week.

Figure 8a and Figure 8b represent Wellness level of four subject houses on a particular test day. Figure 8a shows the Ϣ_1_ went below 0.5 in subject house two; that day the elderly occupant opened the main entry door and forgot to close it. From Figure 8b, the Ϣ_2_ went below in subject house three; that day the elderly person slept more than usual because last night the elderly person had a visitor in the night, which disturbed their sleep. These explanations give an overview of dynamic Wellness determination and anomaly detection as a function of Ϣ_1_ and Ϣ_2_. Hence, depending on the level of Wellness value, the intensity of the alert can be determined. During an anomaly, the alert message would be generated. In the course of attentive memo production, primarily the audio alarm is actuated in the household. This kind of alert message is sent to notify that the elderly person needs to improve certain routines or conduct in order to stay well. In case the elderly person does not turn-off the audio alarm, the alert would be triggered by the care provider or a corresponding authority. Moreover, a care provider and healthcare service provider have access to ADL and well-being monitoring through the website.

The performance characteristics of upgraded Wellness parameters examined through the sensory activations of 43 weeks from four households. Household-two recorded forty-four cautionary messages (highest among all four households) via an upgraded Wellness time series model. The cautionary messages comprise ultimate inactive use of a double-bed (7), ultimate inactive use of a dining armchair (10), ultimate inactive use of the sofa (10), and ultimate inactive application of the E & E household stuff or machinery (17).

The cause of several alarms was an ultimate inactive interval represented by Wellness function parameter (Basic) Ϣ_1_. The inhabitant was out of the home to visit relatives in the town and forgot to update the Wellness System. On the other hand, for the same situations, Upgraded Ϣ_1,_ initiated less alert messages.

The Wellness index (Basic Ϣ_2_) from dining armchair, sofa, and double-bed caused twenty-seven alert messages. However, Wellness function (Upgraded Ϣ_2_) generated significantly less cautionary memos to double bed (3), dining armchair (5), and sofa (5). With the dynamic observation consideration of Wellness, the machine learning model attained a decrease in the alerts. Additionally, this advancement in Wellness time-series pushed the threshold limit to minimize the false positives (fake alerts). False positives reduced by 53.70%.

The investigation of upgraded Wellness function and assessment of Basic Wellness parameters have been shown in Table 3. The improvement in performance of anomaly detection is also a function of the sensor data fusion approach. For household 1 and 3, the Basic Wellness time series model shows an ultimate active period of taking food crossed the boundary and into inconsistency. Nevertheless, conferring to (Upgraded Ϣ_2_), the elderly had a friend for dinner. They were chatting during the meal and took extra time for the eating activity. In household 2, the was inconsistency detected by both Basic and upgraded Wellness time series; the elderly occupant was unhealthy and sleeping longer. Moreover, in another activity from elderly household 2, the Basic Wellness time-series failed to recognize that the over-usage of the toilet was caused by the guest, and Upgraded Wellness time-series discovered the presence of a guest. Whereas for household 4 from the Table 3, it shows the irregularity and over-usage of the toilet from both Basic and Upgraded Wellness time-series, since the sensing system did not record any guest or visitor. The following new annotations have been used: regular (RL), Anomaly (AL), subject house (Sub). Ϣ_2_ Basic* and Ϣ_2_ Upgraded* were evaluated exclusively when the Definite period was more than the ultimate period.

Table 4 shows the values of TIN and TN; the values were measured via derived equations. TIN and TN values lead to Wellness grades and remarks. A one thumb rule had been adopted in the present Smart Aging system—whenever an anomaly detected a sub-activity or activity that did not trigger an alarm, please check that event with multi-sensor data fusion to avoid a false positive. From the observation of the above table records, it is detected that only subject house 4 was free from an anomaly; on the other hand, the rest of the subject houses had anomaly forecasting tags. From subject 1 and 2, they had over-usage of toilet activities, but both had different causes of an anomaly. In the case of subject 1, it was upset stomach, whereas subject 2 had a visitor at home. Additionally, due to the visitor, subject 2 was sitting for more time in a dining armchair while eating, and that caused the anomaly of the armchair over-usage. Subject 3 was in the unhealthy condition that caused him to sleep a lot and eat less.

The performance evaluation of the modeled novel Wellness classifier is recorded through the confusion matrix. Table 5 gives the annotation of ADL’s. For the performance evaluation of the implemented system, a total of fourteen activities have been chosen and tested.

B1 is the bedtime activity and the its parameters are as follows: Sensitivity (0.9911), Specificity (0.9985) Precision (0.9813), Negative Predictive Value (0.9993) False Positive Rate (0.0015), False Discovery Rate (0.0187) False Negative Rate (0.0089), Accuracy (0.9980), F1 Score (0.9862), Correlation Coefficient (0.9851).

B2 is the entry into the home and the evaluation parameters for it are as follows: Sensitivity (0.9789), Specificity (0.9984) Precision (0.9804), Negative Predictive Value (0.9983), False Positive Rate (0.0016), False Discovery Rate (0.0196), False Negative Rate (0.0211), Accuracy (0.9970), F1 Score (0.9797), Correlation Coefficient (0.9780).

B3 is the exit activity and the performance evaluation parameters for it are as follows: Sensitivity (0.9891), Specificity (0.9991), Precision (0.9891), Negative Predictive Value (0.9991), False Positive Rate (0.0009), False Discovery Rate (0.0109), False Negative Rate (0.0109), Accuracy (0.9984), F1 Score (0.9891), Correlation Coefficient (0.9882).

B4 is the usage of water cattle and the evaluation parameters for it are as follows: Sensitivity (1.0000), Specificity (0.9992), Precision (0.9896), Negative Predictive Value (1.0000), False Positive Rate (0.0008), False Discovery Rate (0.0104), False Negative Rate (0.0000), Accuracy (0.9992), F1 Score (0.9948), Correlation Coefficient (0.9944).

B5 is the entertainment activity, which is measured through TV usage. The testing parameters are given as follows: Sensitivity (1.0000), Specificity (1.0000), Precision (1.0000), Negative Predictive Value (1.0000), False Positive Rate (0.0000), False Discovery Rate (0.0000), False Negative Rate (0.0000), Accuracy (1.0000), F1 Score (1.0000) Correlation Coefficient (1.0000).

B6 is the food preparation activity, which is measured by the sensory activation E & E appliance usage. The measurement parameters for it are as follows: Sensitivity (0.9652), Specificity (0.9980), Precision (0.9748), Negative Predictive Value (0.9972), False Positive Rate (0.0020), False Discovery Rate (0.0252), False Negative Rate (0.0348), Accuracy (0.9956), F1 Score (0.9700), Correlation Coefficient (0.9676).

B7 is the activity of eating, and the sensory data belongs to the dining table and dining chair usage. The parameters for it are as follows: Sensitivity (1.0000), Specificity (0.9964), Precision (0.9508), Negative Predictive Value (1.0000), False Positive Rate (0.0036), False Discovery Rate (0.0492), False Negative Rate (0.0000), Accuracy (0.9966), F1 Score (0.9748), Correlation Coefficient (0.9733).

B8 is the use of microwave oven, and the parameters are as follows: Sensitivity (1.0000), Specificity (1.0000), Precision (1.0000), Negative Predictive Value (1.0000), False Positive Rate (0.0000), False Discovery Rate (0.0000), False Negative Rate (0.0000), Accuracy (1.0000), F1 Score (1.0000), Correlation Coefficient (1.0000).

B9 is the usage of the refrigerator, measured by the sensory data of door opening and closing. The parameters are as follows: Sensitivity (1.0000), Specificity (1.0000), Precision (1.0000) Negative Predictive Value (1.0000), False Positive Rate (0.0000), False Discovery Rate (0.0000), False Negative Rate (0.0000), Accuracy (1.0000), F1 Score (1.0000), Correlation Coefficient (1.0000).

B10 is cleaning kitchen, dishes, usage of wash basin activities. The parameter are as follows: Sensitivity (0.9746), Specificity (0.9981), Precision (0.9751), Negative Predictive Value (0.9980), False Positive Rate (0.0019), False Discovery Rate (0.0249), False Negative Rate (0.0254), Accuracy (0.9963) F1 Score (0.9748), Correlation Coefficient (0.9728).

B11 is the usage of shower and it is recognized by the opening and closing of the shower door. The parameters are as follows: Sensitivity (0.9791), Specificity (0.9978), Precision (0.9728) Negative Predictive Value (0.9984), False Positive Rate (0.0022), False Discovery Rate (0.0272), False Negative Rate (0.0209), Accuracy (0.9965), F1 Score (0.9759), Correlation Coefficient (0.9740).

B12 is the toilet usage and it is detected with the sensory data of force sensor deployed over the toilet seat and movement sensors. The parameters for it are as follows: Sensitivity (0.9725), Specificity (0.9983), Precision (0.9788), Negative Predictive Value (0.9978), False Positive Rate (0.0017), False Discovery Rate (0.0212), False Negative Rate (0.0275), Accuracy (0.9965), F1 Score (0.9756), Correlation Coefficient (0.9737).

B13 is the usage of computer desktop and the parameters are as follows: Sensitivity (1.0000), Specificity (1.0000), Precision (1.0000), Negative Predictive Value (1.0000) False Positive Rate (0.0000), False Discovery Rate (0.0000), False Negative Rate (0.0000), Accuracy (1.0000), F1 Score (1.0000), Correlation Coefficient (1.0000).

B14 is the relaxing activity monitored by the occupancy over the sofa set. The parameters for it are as follows: Sensitivity (0.9430), Specificity (1.0000), Precision (1.0000), Negative Predictive Value (0.9957), False Positive Rate (0.0000), False Discovery Rate (0.0000), False Negative Rate (0.0570), Accuracy (0.9960), F1 Score (0.9706), Correlation Coefficient (0.9690).

The activity recognition done with the sensory input of the E & E appliance monitoring sensory unit has a significantly better score as compared to non-E & E sensory units, as the E&E sensory-unit-based activities do not require the sensor data fusion and other sub-module for the activity recognition. On the other hand, the relaxing and eating activities have recorded the poorest score in those 14 activities, because these two activities use the sensory input of force sensors. Force sensors sometimes went in saturation when deployed over the Dining chair, Table, and Sofa set. 

The overall scores for the selected 14 activities are as follows: Sensitivity (0.9852), Specificity (0.9988), Precision (0.9887), Accuracy (0.9974), F1 score (0.9851), Correlation Coefficient (0.9144), False Negative Rate (0.0130)

**Figure 9:**
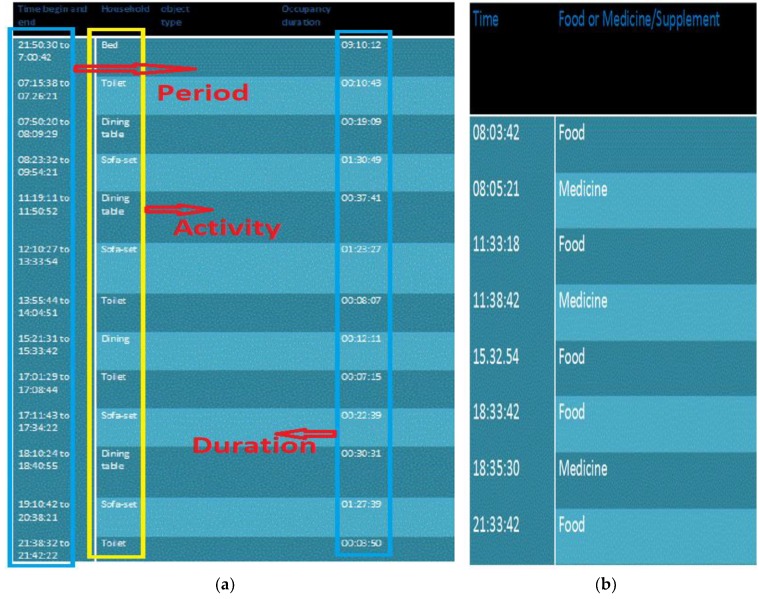
(**a**) The activities during the course of the day. (**b**) The activities of taking medication and food during the course of the day.

A web-based Intelligent Aging framework had been produced. The data was gathered and treated through sensor data fusion calculations for the decision making. At last, data was transferred to the web link; the data over the internet was opened to a validated client by means of enlisted email id and secret word. The initial ADL recognition was completed by the heterogeneous sensor network; the push button indicator data did not apply. Nonetheless, there are a couple of exercises that the framework does not recognize effectively, for example, taking supper or pharmaceuticals on time. For this sort of exercises, either of the frameworks utilize prominent checking routes, for example, camera, wearable sensors, or go with a parental figure every minute of every day, which isn’t attainable and economical [4,5]. Moreover, in the current investigation, the push button indicator information was applied to crosscheck the ADLs produced via the information secured from diverse sensing units. Figure 9a,b indicate previews in the Wellness framework website. This site comprises the information of recent ADLs. In order to check the observing historical ADLs from a specific date, the subject needs to choose a date and time. Figure 9a demonstrates the data on the non-electrical machine use. It demonstrates resting, eating, and latrine exercises: for instance, on August 30, a tenant went to the toilet in the morning for 00:10:43 (hours:minute:seconds). Figure 9b displays the observing of nourishment and medication of a tenant. It shows that the inhabitant was taking a drug soon after the food and daily three events of medication were recorded.

Table 6 presented the comparative overview of the Wellness model with other most significant, recent, and peer-reviewed research works. The performance of the existing system is either average or very limited as compared to our offered research work. There are the following vital considerations and methodologies with support from the optimum Wellness system performance:

1. The Wellness Model is an innovative methodology that classifies events inside definite temporal thresholds of activity initiation. The Wellness system recommended an accumulative overlapped-defined sized sliding window procedure, which sections the flooding sensor sub-activities into adaptable interval distances to assist the discovery of activities in an appropriate approach. Events are categorized as either prompt or periodical; an event based attitude can then be implemented for recognition of discrete events. The Wellness system was cross verified and examined by the subject´s inputs.
2. Current research work accumulates sensing data, modeled ADLs, mines appropriate arithmetical features, and employs supervised machine learning to forecast well-being scores for healthcare applications.
3. We offered dynamic frequent feature extraction and a forecast model based on Wellness indices time series modeling. Preliminary trials presented that one-day timespan was best for data mining, however, we constructed the framework to function on any dramatic time. Test outcomes have confirmed the usability of the research methodology to appropriately distinguish various household object, appliance usage, and offer long or short term trends accordingly.

## 5. Conclusions and Future Work

The well-being recognition performed by producing the ADLs was based on the movement inside the home and heterogeneous object usage. The smart Aging system applied the sensor data fusion approach to improve the anomaly detection over the large heterogeneous sensory data. These ADL recognition and well-being pattern generations distinguished any peculiarity change in the routine. Through Advanced believe model the mysterious and flawed behavior of sensing data was recognized, the model addressed the underfitting and overfitting. Wellness sensor data fusion model used sensor data from multiple contexts, and processed it on the basis of priority and event. The present Novel Wellness Indices Modeling and Detection Methodology processed the behavioral pattern from multiple sensor modalities with the least fake detection rate. Anomaly detection and forecasting methodology considered the cyclic variations and random day occasions. The Wellness Model processes data sampled at diverse rates and multi-modality sensory data with minimal and ordinal standards structured in hierarchies, not like numerous machine learning procedures that deal only with numeric entities.

Concerning pattern inconsistency, the assessment revealed that the Wellness model finds sensible, manifold, and distinct-length temporal values for every event. The benefit of Wellness based AAL is that it picks up the period from the timestamp in a solo scan uninterruptedly from the data stream of annotations without the requirement to reinstruct at what time fresh annotations developed. This distinctive merit permits it to include context-aware deviations for every trend as they are cultured and to adjust to variations over interval without reorientation. Wellness smart aging system presented that without including contextual features, the trends fail to reveal the ADL fluctuations. In such cases, irregularities can be discovered that don’t relate to genuine deviations of the ordinary everyday practice. Wellness model defines contextual feature statistics during the analysis and learning stages, discovering deviations in routine and routine flexibility for every contextual feature. Context-aware trends and sequences could intimate dissimilarities in ADLs and enhance learning.

The framework was executed in four distinct households; two of them were exceptionally timeworn households. The framework had been outlined in a way that the elderly person was free from serious maintenance except for the power supply and physical damage. The smart aging system offered the average anomaly detection rate for 300 days and four elderly houses at 98.17%. The performance of evolution parameters for the selected 14 activities were as follows: Sensitivity (0.9852), Specificity (0.9988), Precision (0.9887), Accuracy (0.9974), F1 score (0.9851), Correlation Coefficient (0.9144), False Negative Rate (0.0130). Accuracy does not give an overall picture of the F1 score.

One of the major reasons behind the degradation in the F1 measure is noise and interference error. There is a need to filter data noises prior to the lifestyle modeling process. Two types of data noises should be filtered. First, anomaly behavioral patterns that are generated by the visitor. Second, anomaly sensor activations (data outlier) that are generated by the faulty sensor. To filter the former, CFM (Collaborative Filtering Model) with spatial states Gaussian Distribution (GD) would be a good methodology to discover routine behavioral patterns generated by the single resident, apart from visitors and pets. The assessment of the execution of frameworks on these smart aging datasets and the variety in their execution obviously underlines the significance of having the highest quality level which truly accomplishes the best quality, i.e., which are free of mistakes. This is by inferring hidden latent states that represent the resident’s identity and habit. To filter the latter, the Advanced Belief Model is employed to filter data outliers from a faulty sensor, but it is not sufficient. We henceforth see this work as a first venturing stone in a bigger plan relating to enhancing the appraisal of the execution of a Smart Aging system.

Processed data was uploaded to the website with the help of the internet, and decision making information was produced. The uploaded data and decision-making information are accessible through the authentic login ID and password by the caregiver, healthcare professional, police, or any other official. In a few fields, those specifically related with the components of wellness (including wellbeing, movement, and emotion), some future examinations among experts (e.g., health specialists, medical attendants, analysts, and so on.) will profit from further enhancements of this model. Concerning current innovation and our past tasks, there is solid proof that the bits of knowledge and arrangements introduced are conceivable. The measurements exhibited have observable commitments to the prosperity of everybody, and we unequivocally trust those future activities established on this model will permit enhancement of the personal satisfaction of the elderly. A few perspectives were discussed with doctors, which presented to us a few viewpoints to represent in future executions. Besides, the elements of wellbeing and human-information technology communication strategies proposed are expected for the execution of “progressively characteristic” interfaces, which can adequately improve the well-being of the older adults.

For future work, we are intending to upgrade the Wellness indices model and present distributed learning in large data processing from different houses in near real time. This will help well-being applications to speedily take activities, for example, sending an alarm to patients or care suppliers. Besides, we are intending to construct a wellbeing cosmology model to naturally outline machines for potential ADL. This implies that we can proficiently prepare the framework and precisely distinguish human ADL.

Summary Points:

(1) What is Already Known
  - Wearable and Camera Based ADL monitoring for the individual living alone in the indoor urban environment.
  - Subject’s input-based data annotation.
  - Little research exists regarding the development of heterogeneous Wireless Sensor Networks and sensor data fusion in near real-time on behavioral pattern generation, workflow, and the parameters that lead to the improve the life span.
  - Most of the research is limited to the laboratory, and it becomes unfeasible for the uncontrolled home environment.
(2) What This Study Adds to Our Knowledge
  - Addition of Cutting-edge Wireless Sensing and Information Technology for the Wellness evaluation of an occupant living in an assisted environment.
  - Precise Wellness measurement from streaming data with the novel offering of the Wellness Indices Model.
  - The incorporated structure of the Wellness Protocol offers the functionality of recording the occupant’s movement in real time, and the usage of different household objects.
  - Recognition of ADLs for behavioral pattern generation, behavioral forecasting, and anomaly detection with the least possible fake alert.

## Figures and Tables

**Table 1:** Activity Annotation for 24 h.

Unique Node ID	Household Stuff Connected	Sensor Applied	Timestamp	Usage	Activity Annotation
00FR1	Bedstead	Force	21:07:33 2016-09-10 Start SL 07:48:10 2016-09-11 End SL	10 h 40 min 37 s	Sleeping
00FR2	Toilet seat	Force	07:58:34 2016-09-11 Start TL 08:07:12 2016-09-11 End TL	8 min 38 s	Toilet
00A1, 00A2, and 00A3	Electric stove, Grill, and Rice Cooker	E & E monitoring unit	08:17:23 2016-09-11 Start BF 08:32:24 2016-09-11 End BF	15 min 1 s	Breakfast Cooking
00CN1	Shower Door	Contact	09:22:44 2016-09-11 Start SW 09:35:21 2016-09-11 End SW	12 min 37 s	Shower
00A1, 00A4, 00A5, and 00A6	Electric stove, Grill, Rice Cooker, and Refrigerator	E & E monitoring unit	10:24:17 2016-09-11 Start LN 11:17:40 2016-09-11 End LN	53 min 23 s	Lunch Cooking

**Table 2:** Number of sensor activation and activity detection for four different houses equipped with hydrogenous sensing units.

Label	Household 1	Household 2	Household 3	Household 4
Sensing units (Nodes)	37	32	37	29
Weeks monitored	43	43	43	43
Sensory triggering	693,705	605,758	725,671	650,521
ADLs uncovering	14,067	11,987	15,004	11,541

**Table 3:** Upgraded Wellness Function vs. Basic Wellness Function for recognizing ADLs.

Sub	Sensor and Location	Activity	Ultimate- Active Interval (Sec)	Lowest-Active Interval (Sec)	Definite Real Interval (Sec)	Ϣ1, Basic	Ϣ2, Basic *	Activity Detection via Basic Functions	Ϣ1, upgraded	Ϣ2, upgraded *	Activity Detection via upgraded Functions
#1	Force-Bed, Movement-Bedroom	Sleeping	32,128	25,712	27,491	0.85	RL	RL	0.89	RL	RL
	**Force-Dinning Armchair, Movement -Dining Area**	**Taking ** **Food**	**5532**	**3829**	**2143**		**0.53**	**AL**		**0.77**	**RL**
	Force -Toilet Seat, Movement-Toilet Door	Toilet/Latrine	1835	1321	1521		RL	RL		RL	RL
	Force-Sofa Movement-Living Room	Calming	2012	1092	1238			RL			RL
	Contact/Force Sensor-Shower Door Movement-Bathroom	Shower/Personal Hygiene	1627	1170	1532			RL			RL
	E & E Sensor-TV	TV	3852	2438	2842			RL			RL
#2	**Force-Bed, Movement-Bedroom**	**Sleeping**	**30,200**	**22,245**	**33,212**	**0.77**	**0.54**	**AL**	**0.83**	**0.66**	**AL**
	Force-Dinning Armchair, Movement -Dining Area	Taking Food	4321	3213	3015		RL	RL		NA	RL
	**Force -Toilet Seat, Movement-Toilet Door**	**Toilet/** **Latrine**	**1732**	**1245**	**2223**		**0.52**	**AL**		**0.69**	**RL**
	Force-Sofa Movement-Living Room	Calming	1823	1138	1426		RL	RL		RL	RL
	Contact/Force Sensor-Shower Door Movement-Bathroom	Shower/Personal Hygiene	1578	1262	1492			RL			RL
	E & E Sensor-TV	TV	3647	2745	2984			RL			RL
#3	Force-Bed, Movement-Bedroom	Sleeping	28,431	20,245	27,212	0.80		RL	0.89		RL
	**Force-Dinning Armchair, Movement -Dining Area**	**Taking Food**	**3981**	**2834**	**4413**		**0.57**	**AL**		**0.79**	**RL**
	Force -Toilet Seat, Movement-Toilet Door	Toilet/Latrine	1838	1341	1530		RL	RL		RL	RL
	Force-Sofa Movement-Living Room	Calming	1749	1369	1393			RL			RL
	Contact/Force Sensor-Shower Door Movement-Bathroom	Shower/Personal Hygiene	1405	1136	1303			RL			RL
	E & E Sensor-TV	TV	3729	3021	3213			RL			RL
#4	Force-Bed, Movement-Bedroom	Sleeping	30,261	27,492	26,492	0.83	RL	RL	0.91		RL
	Force-Dinning Armchair, Movement -Dining Area	Taking Food	3620	2785	3329			RL			RL
	**Force -Toilet Seat, Movement-Toilet Door**	**Toilet/Latrine**	**1839**	**1384**	**2318**		**0.48**	**AL**		**0.65**	**AL**
	Force-Sofa Movement-Living Room	Calming	1640	1243	1620		RL	RL		RL	RL
	Contact/Force Sensor-Shower Door Movement-Bathroom	Shower/Personal Hygiene	1537	1138	1430			RL			RL
	E & E Sensor-TV	TV	3124	2481	2647			RL			RL

**Table 4:** Well-being detection and anomaly forecasting on the basis of Wellness parameters.

	ADL	SID	Ϣ_1_	Ϣ_2_	Forecasting for an Upcoming Week					Actual-Duration (Sec)	Status
					Max-Time (Sec)	Min-Time (Sec)	α	δ	Γ		
#1	Force-Bed, Movement-Bedroom	Sleeping	0.83	0.842	27,483	24003	0.200	0.120	0.421	25,470	RL
	Force-Dinning Armchair, Movement -Dining Area	Taking Food		0.932	4743	3216	0.140	0.058	0.490	3324	RL
	**Force-Toilet Seat, Movement-Toilet Door**	**Toilet/Latrine**		**0.482**	**1975**	**1347**	**0.030**	**0.0580**	0.700	**2564**	**AL**
	Force-Sofa, Movement-Living Room	Calming		0.883	1487	1256	0.030	0.300	0.500	1409	RL
	Contact/Force Sensor-Shower Door Movement-Bathroom	Shower/Personal Hygiene		0.965	1765	1437	0.200	0.320	0.700	1432	RL
	E & E Sensor-TV	TV		0.863	3689	2864	0.059	0.200	0.300	3257	RL
#2	Force-Bed, Movement-Bedroom	Sleeping	0.795	0.810	27,492	21,394	0.030	0.120	0.600	26,408	RL
	**Force-Dinning Armchair, Movement -Dining Area**	**Taking Food**		**0.507**	**4923**	**3028**	**0.200**	**0.050**	**0.400**	**6322**	**AL**
	Force -Toilet Seat, Movement-Toilet Door	Toilet/Latrine		**0.423**	**1829**	**1449**	**0.048**	**0.370**	**0.540**	**2302**	**AL**
	Force-Sofa Movement-Living Room	Calming		0.785	1420	1124	0.020	0.400	0.350	1294	RL
	Contact/Force Sensor-Shower Door Movement-Bathroom	Shower/Personal Hygiene		0.842	1530	1204	0.100	0.540	0.530	1420	RL
	E & E Sensor-TV	TV		**0.40**	**4502**	**3954**	**0.100**	**0.070**	**0.400**	**1020**	**AL**
#3	Force-Bed, Movement-Bedroom	Sleeping	0.846	**0.500**	**31,289**	**25,491**	**0.030**	**0.560**	**0.605**	**36,506**	**AL**
	**Force-Dinning Armchair, Movement -Dining Area**	**Taking Food**		**0.490**	**4839**	**3429**	**0.300**	**0.460**	**0.170**	**1002**	**AL**
	Force -Toilet Seat, Movement-Toilet Door	Toilet/Latrine		0.820	1509	1145	0.350	0.330	0.700	1329	RL
	Force-Sofa Movement-Living Room	Calming		0.889	1530	1239	0.120	0.300	0.500	1430	RL
	Contact/Force Sensor-Shower Door Movement-Bathroom	Shower/Personal Hygiene		0.943	1620	1307	0.100	0.459	0.300	1540	RL
	E & E Sensor-TV	TV		0.920	3309	2845	0.400	0.340	0.600	2984	RL
#4	Force-Bed, Movement-Bedroom	Sleeping	0.864	0.863	29,042	25,302	0.150	0.300	0.400	27,404	RL
	**Force-Dinning Armchair, Movement -Dining Area**	**Taking Food**		**0.922**	**3942**	**2948**	**0.300**	**0.530**	**0.350**	**3720**	**RL**
	Force -Toilet Seat, Movement-Toilet Door	Toilet/Latrine		0.824	2450	1750	0.250	0.300	0.740	1730	RL
	Force-Sofa Movement-Living Room	Calming		0.842	1730	1284	0.300	0.350	0.600	1630	RL
	Contact/Force Sensor-Shower Door Movement-Bathroom	Shower/Personal Hygiene		0.945	1430	1039	0.200	0.300	0.400	1240	RL
	E & E Sensor-TV	TV		0.864	3102	2503	0.400	0.300	0.600	2830	RL

**Table 5:** Annotation used.

S.No.	Type of Activity
B1	Bedtime
B2	Entry
B3	Exit
B4	Water cattle
B5	Television
B6	Food preparation-cooking
B7	Dining table-Eating chair
B8	Microwave Oven
B9	Fridge usage (by the door)
B10	Wash basin/kitchen hygiene/dishwashing
B11	Shower
B12	Toilet usage
B13	Computer use
B14	Sofa-relax

**Table 6:** Comparative overview of present work with existing ADLs monitoring and forecasting system.

TITLE	CATEGORY OF AAL APPLICATION	DATA COLLECTION	SET OF ACTIVITIES	TYPE OF MACHINE LEARNING ALGORITHM	RELIABILITY AND PERFORMANCE PARAMETERS, MERITS OF ANNOTATION, ACTIVITY DETECTION, AND FORECASTING
PROJECT					
COBRA (CUMULATIVELY OVERLAPPING WINDOWING APPROACH FOR AMBIENT RECOGNITION OF ACTIVITIES) [37]	BEHAVIORAL RECOGNITION	USES CASAS DATASET. MORE THAN 6500 ACTIVITIES RECOGNIZED FROM IT.	SLEEPING, MEAL PREPARATION, RELAX, HOUSEKEEPING, EATING, LEAVE HOME, ENTER HOME, WORK	SLIDING WINDOW TECHNIQUE	RECOGNITION ACCURACY (0.821) AND RECALL (0.89)
AUTOMATED FEATURE ENGINEERING [38]	ACTIVITY DETECTION	OPEN SOURCE DATASETS OF DIFFERENT RESEARCH GROUPS WITH THE APPLICATION OF WEARABLE SENSORS	WALKING, STANDING, SITTING, VACUUMING, SWEEPING	SVM, NB, AND KNN	ACTIVITY DETECTION PRECISION (0.83) RECALL (0.80)
MINING HUMAN ACTIVITY PATTERNS FROM SMART HOME BIG DATA [39]	ACTIVITY DETECTION AND FORECASTING	400 MILLION SENSORS ACTIVATIONS	WATCHING TV, COOKING, USING COMPUTER, PREPARING FOOD AND CLEANING DISHES OR CLOTHES	BAYESIAN NETWORKS	PREDICTION ACCURACY (0.80)
LAPLACE [40]	ACTIVITY DETECTION AND FORECASTING	OPEN SOURCE DATASETS OF DIFFERENT RESEARCH GROUPS	WAKE UP, SHOWER, EAT, GOING OUT, RELAX, COOK	FREQUENT SEQUENTIAL PATTERN MINING	DID NOT mention THE PARAMETER VALUE, THE PERFORMANCE WAS AVERAGE
AGACY MONITORING [41]	ACTIVITY DETECTION	SENSING SYSTEM DEVELOPED	PREPARING FOOD, EAT, REST, DISHWASHING, WAKEUP	ONTOLOGICAL MODELING, SEMANTIC REASONING, AND DEMPSTER SHAFER THEORY	ACTIVITY DETECTION PRECISION (0.91) F1 SCORE (0.87) AND RECALL (0.83)
CONTEXTUALIZED BEHAVIOR PATTERNS [42]	BEHAVIORAL RECOGNITION AND FORECASTING	CASAS DATASETS OF 193 DAYS USED	MEAL PREPARATION, SLEEPING, WASH DISHES, WORK, ENTER HOME, LEAVE HOME, TOILET, HOUSEKEEPING, RELAX, EATING	CONTEXTUALIZED PREFIX-TREE	DID NOT claim ANNOTATION AND ADL RECOGNITION MERIT VALUES. THE FORECASTING PRECISION VALUES (0.392) AND RECALL VALUE (0.41)
AGING IN PLACE BY CHRITIAN DEBES [43]	BEHAVIORAL RECOGNITION	DATA WAS COLLECTED FROM TWO HOUSEHOLDS WITH MORE THAN 1000 ACTIVITY INSTANCES	PERSONAL HYGIENE, SLEEP WORK, MEAL PREPARATION, WATCH TV, SLEEP, SHOWERING	SVM, HMM AND FISHER KERNEL LEARNING (FKL)	THE ADL DETECTION CLASS AVERAGE ACCURACY FOR FKL (0.71), HMM (0.69) AND SVM (0.68)
ONLINE DAILY HABIT MODELING AND ANOMALY DETECTION (ODHMAD) MODEL [44]	BEHAVIORAL AND ANOMALY DETECTION	OBTRUSIVE AND UNOBTRUSIVE SENSING SYSTEM	MOVEMENT, OPEN-CLOSE STATES OF DOOR/WINDOW, FLUSH TOILET, USE OF ELECTRICAL DEVICES, TAKE SHOWER, WASH HAND, FALLS, EATING	ONLINE ACTIVITY RECOGNITION (OAR)	ANOMALY PRECISION (0.78), FALSE ALARM RATE (0.21) AND MISS DETECTION RATE (0.11)
WELLNESS INDEX MODEL	Behavioral PATTERN GENERATION AND ANOMALY DETECTION	UN-OBTRUSIVE HETEROGENEOUS WIRELESS SENSORS NETWORK	SLEEPING TAKING FOOD TOILET/LATRINE CALMING SHOWER/PERSONAL HYGIENE USE OF APPLIANCES	Novel Wellness Indices Modelling and Detection ALGORITHM	Sensitivity (0.9852), Specificity (0.9988), Precision (0.9887), Accuracy (0.9974), F1 score (0.9851), Correlation Coefficient (0.9144), False Negative Rate (0.0130)
